# Efficient whole-genome sequencing of Monkeypox virus using a novel nuclease-multiple displacement amplification enrichment method

**DOI:** 10.1038/s44298-026-00170-z

**Published:** 2026-01-21

**Authors:** Masayasu Misu, Takeshi Kurosu, Tomoki Yoshikawa, Madoka Kawahara, Kohei Oishi, Masayuki Shimojima, Hideki Ebihara

**Affiliations:** Department of Virology I, National Institute of Infectious Diseases, Japan Institute for Health Security, Musashimurayama-shi, Tokyo Japan

**Keywords:** Biological techniques, Genetics, Microbiology, Molecular biology

## Abstract

Monkeypox virus (MPXV) has a large double-stranded DNA genome (~200 kb), which presents challenges for whole-genome sequencing. Conventional enrichment methods have specific limitations. To address them, we developed a novel enrichment strategy combining nuclease treatment and multiple displacement amplification (MDA), along with terminal PCR to compensate for the reduced read depth at the genome termini. When applied to 18 historical isolates, this method yielded more than 96% MPXV-specific reads, enabled complete genome assembly, and demonstrated reproducibility and robustness in the phylogenetic analysis. It successfully sequenced low-titer samples (up to a Ct value of 33.5), suggesting a potential performance comparable to PCR-based methods, and demonstrated broad applicability to other Orthopoxviruses, such as Cowpox and Ectromelia viruses. The nuclease–MDA method is cost-effective, avoids the issue of primer/probe mismatches, and is applicable to low-titer samples. This strategy offers a competitive and alternative for MPXV and related Orthopoxviruses genomic surveillance and fundamental research.

## Introduction

Monkeypox virus (MPXV), the causative agent of mpox, has historically circulated in Central and West Africa^1–3^. With recent changes in global travel patterns and waning immunity due to the cessation of smallpox vaccination, it has become more widespread^3,4^. The 2022 outbreak of clade IIb lineage B.1 marked a shift in transmission dynamics, with sustained human-to-human transmission rather than the traditional zoonotic route^5,6^. This outbreak spread beyond endemic regions and exhibited new dissemination patterns. In early 2024, genomic surveillance identified a previously unrecognized clade, Ib, in the Democratic Republic of the Congo, which has since spread to neighboring countries, with imported cases reported outside Africa^7–9^. These developments underscore the need for continuous genomic surveillance to track MPXV evolution, support epidemiological investigations, and inform public health responses through accurate and scalable genome sequencing^10–12^.

MPXV has a large, linear double-stranded DNA genome (~200 kb), posing technical challenges for sequencing. When whole-genome sequencing (WGS) of MPXV is performed using shotgun metagenomic sequencing with clinical specimens or isolated viral strains^13,14^, the resulting reads inevitably include a substantial number of reads originating from the host genome. Consequently, the proportion of MPXV-derived reads relative to the total number of reads was low. Achieving high-accuracy WGS across the entire MPXV genome therefore necessitates the acquisition of a large number of reads using high-throughput sequencers, consequently imposing a significant cost burden.

Viral genome enrichment prior to sequencing is essential to enhance the accuracy and cost-effectiveness of MPXV WGS. Existing enrichment strategies for MPXV WGS can be broadly classified into two major categories. The first is targeted enrichment approaches. This category includes Hybridization Capture, which concentrates the target sequence by designing biotin-labeled probes complementary to the MPXV genome, mixing them with sample DNA for specific binding (hybridization), and retrieving the complex using magnetic beads^15^. It also includes PCR-based enrichment (Multiplex tiling PCR)^7,16^, and a multiple displacement amplification (MDA) method combining Phi29 polymerase with a few specific primer sets (i.e., 6 octamer primers)^17^.

While the MDA-based approach significantly reduces the number of primers, thereby reducing the risk of primer mismatches, Hybridization Capture and especially Tiling PCR rely on the sequence stability of probes or primers. Given the large MPXV genome and reported mutations/recombinations, probe- or primer-mismatches present the risk of coverage dropout or non-uniform amplification across the entire genome.

The second category consists of nuclease-pretreated metagenomic approaches^18^. This method involves selective lysis of host cells, degradation of host-derived free DNA by DNase treatment, and concentration of viral particles, followed by WGS. As this method does not include a genome amplification process, it avoids coverage dropout due to probe or primer mismatches and can enrich MPXV-derived reads to approximately 50% of the total reads.

To improve the efficiency and robustness of MPXV WGS, we developed a new strategy that combined micrococcal nuclease treatment and MDA, along with a terminal PCR step. This method achieves an enrichment efficiency comparable to that of target-specific probe- or primer-based approaches, without relying on them, making it a cost-effective option for routine implementation. MDA can lead to a reduced read depth at the genome termini. To address this drawback and further enhance sequencing accuracy, terminal PCR targeting conserved terminal regions was performed. We used this method to sequence 18 MPXV isolates stored at the National Institute of Infectious Diseases, Japan (NIID), and validated its utility through molecular phylogenetic analysis.

## Results

### Overview of MPXV genome enrichment using nuclease–MDA

**Fig. 1:**
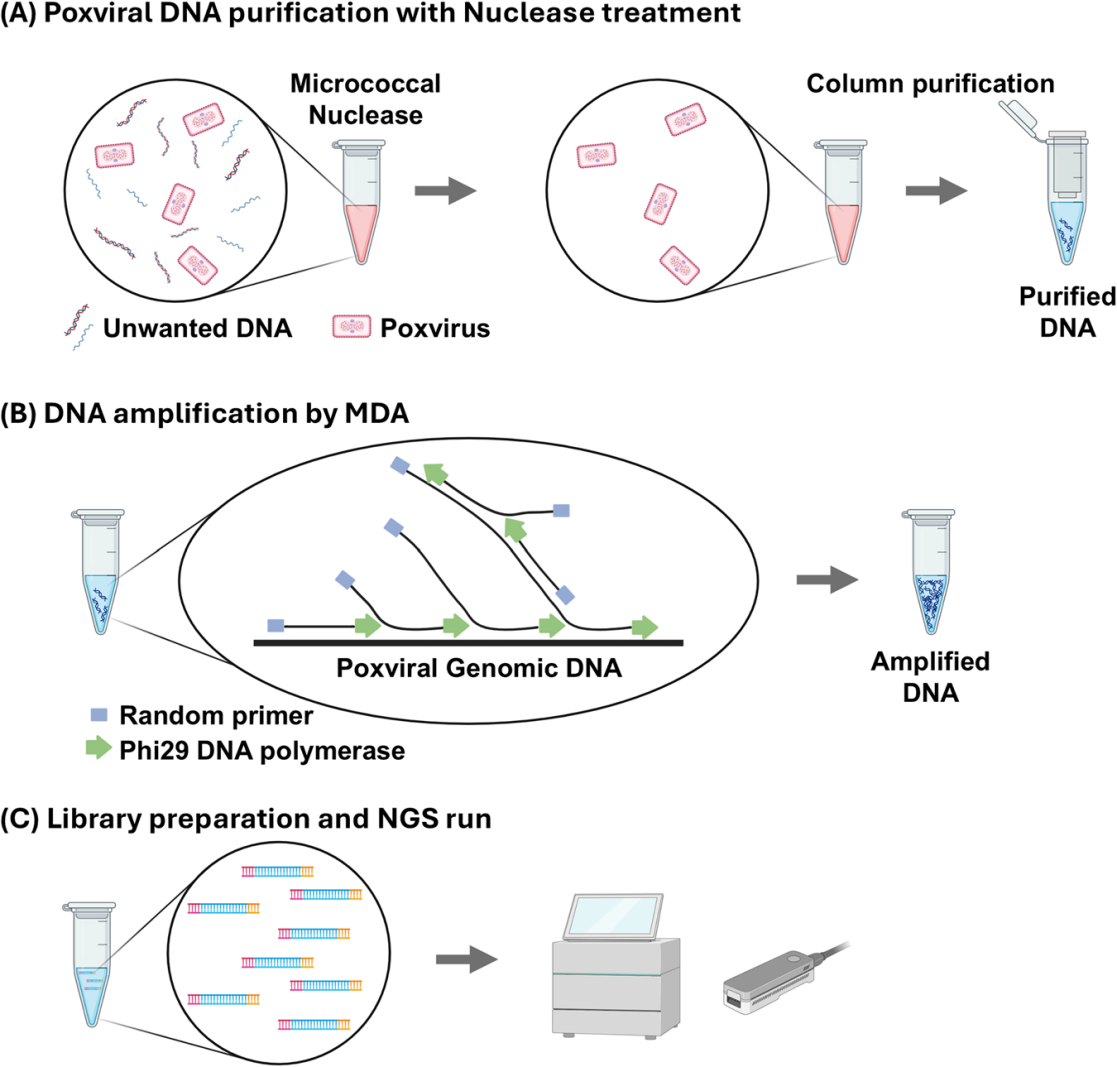
Overview of MPXV genome enrichment using the nuclease–MDA method. An MPXV working stock is treated with micrococcal nuclease to remove unprotected host DNA while preserving viral DNA within intact virions. **A** The DNA is purified using column-based extraction. **B** The purified DNA is amplified using multiple displacement amplification (MDA) with Phi29 polymerase and random primers, enriching MPXV genomic DNA. **C** The enriched DNA is used for library preparation and next-generation sequencing (NGS). The image was created using BioRender.com.

The starting material for the nuclease–MDA method is a working stock of isolated MPXV. Host genomic DNA in the working stock is digested with Micrococcal Nuclease (New England Biolabs [NEB]), while the MPXV genome is protected from degradation by its intact viral envelope (Fig. 1A). The purified viral nucleic acids are subjected to MDA with Phi29 polymerase, enriching the MPXV genome without the need for specific primers or probes (Fig. 1B). The enriched DNA is then used for library preparation and sequencing on either Oxford Nanopore Technologies (ONT) or Illumina platforms (Fig. 1C).

### Effect of nuclease selection on poxvirus genome enrichment

We comparatively evaluated the effectiveness of Micrococcal Nuclease (NEB), OmniCleave Endonuclease (Biosearch Technologies), and DNase I (Promega) in enriching poxvirus genomes using the nuclease–MDA method. A working stock of the vaccinia virus strain LC16m8 (m8), an Orthopoxvirus like MPXV, was used as a model and treated with these nucleases or a mock control. Real-time (q)PCR was used to quantify m8 genome copies (*G9R* gene) as a marker for poxvirus genome enrichment, and RK13 cell genome copies (*GAPDH* gene) to assess host DNA contamination.

**Fig. 2:**
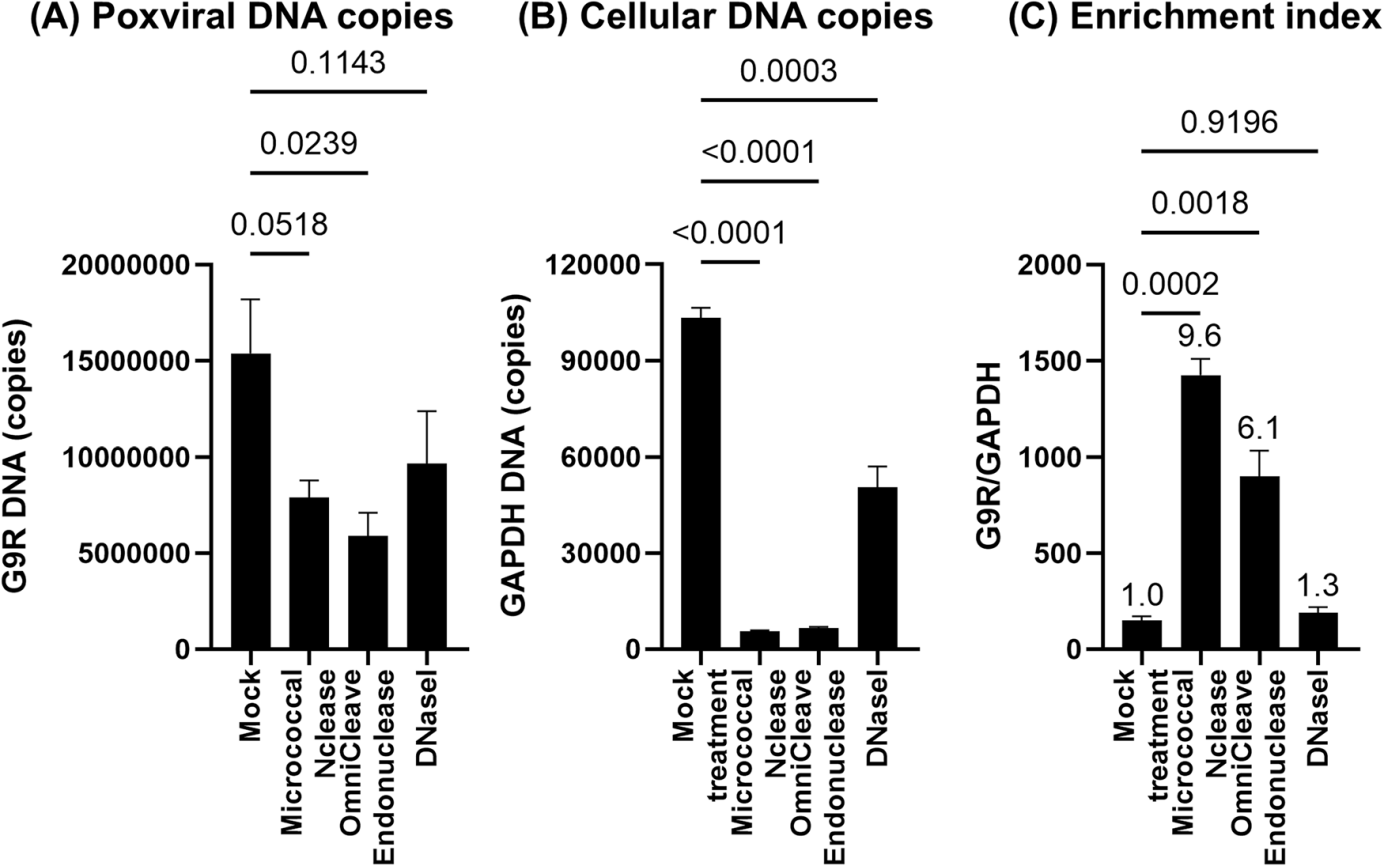
Appropriate nuclease selection is critical for efficient poxvirus genome enrichment. A working stock of m8 was treated with Micrococcal Nuclease, OmniCleave Endonuclease, or DNase I. A mock treatment served as a control. **A** m8 viral genome copies quantified using qPCR targeting the m8 genomic *G9R* gene. **B** Host genomic DNA contamination as assessed using qPCR targeting the rabbit genomic *GAPDH* gene. **C** Enrichment index calculated as the *G9R*/*GAPDH* ratio. Fold increases relative to the mock (set to 1.0) are indicated above the bars. Statistical significance was assessed using one-way ANOVA with Dunnett’s multiple comparison test; *p*-values are shown above the compared groups.

All nuclease treatments slightly reduced m8 genome copy numbers (Fig. 2A) and effectively removed host DNA, with DNase I being the least effective (Fig. 2B). Micrococcal Nuclease preserved the highest number of m8 genome copies while achieving the most significant reduction in host DNA. The enrichment index (*G9R*/*GAPDH* ratio) was the highest for Micrococcal Nuclease (9.6-fold increase, p-value = 0.0002), followed by OmniCleave (6.1-fold, *p* = 0.0018) and DNase I (1.3-fold, *p* = 0.9196) (Fig. 2C). Therefore, the NEB Micrococcal Nuclease was used in subsequent experiments.

### Micrococcal nuclease and MDA synergistically enhance poxvirus genome enrichment

The effect of micrococcal nuclease treatment before MDA on genome enrichment was assessed using a matrix experiment conducted using the m8 working stock with or without micrococcal nuclease treatment and MDA. Nucleic acids were purified from the samples prepared in the matrix experiment, and *G9R* and *GAPDH* copies were quantified using qPCR.

**Fig. 3:**
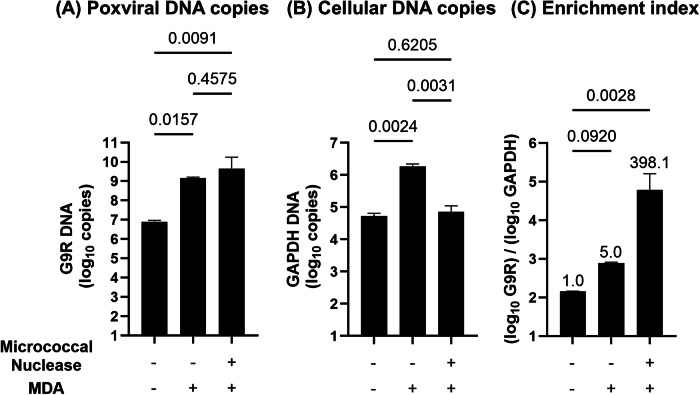
Micrococcal nuclease and MDA act synergistically in poxvirus genome enrichment. An m8 working stock was treated or not with micrococcal nuclease and subjected to MDA (GenomiPhi), followed by nucleic acid purification. **A** m8 genome copies quantified using qPCR targeting the genomic *G9R* gene. **B** Host DNA quantified using qPCR targeting rabbit genomic *GAPDH*. (C) Enrichment index (*G9R*/*GAPDH* ratio). **C** Fold increases relative to the mock (set to 1.0) are shown above the bars. Statistical significance was determined using one-way ANOVA with Tukey’s multiple comparison test; *p*-values are shown above the compared groups.

MDA significantly increased the *G9R* gene copy number by more than 100-fold compared to that in the sample without MDA, whereas micrococcal nuclease treatment did not significantly affect the amplification of *G9R* gene copies (Fig. 3A). However, micrococcal nuclease treatment did drastically reduce *GAPDH* gene amplification by MDA to less than 1/10th of the copy number in the sample without nuclease treatment (Fig. 3B).

The enrichment index, calculated as the ratio of *G9R* to *GAPDH* gene copies, was maximized when both micrococcal nuclease treatment and MDA were applied, with an approximately 400-fold increase compared to that in the absence of both treatments (Fig. 3C).

These findings demonstrate that micrococcal nuclease treatment and MDA synergistically enhance poxvirus genome enrichment through the strong suppression of cellular DNA amplification by nuclease treatment before MDA.

### Effects of various whole-genome amplification (WGA) methods on poxvirus genome amplification bias

The nuclease–MDA method employs the GenomiPhi technology for MDA with Phi29 polymerase and random primers. To assess whether this method provides unbiased poxvirus WGA compared with other WGA methods, we conducted a comparative analysis.

**Fig. 4:**
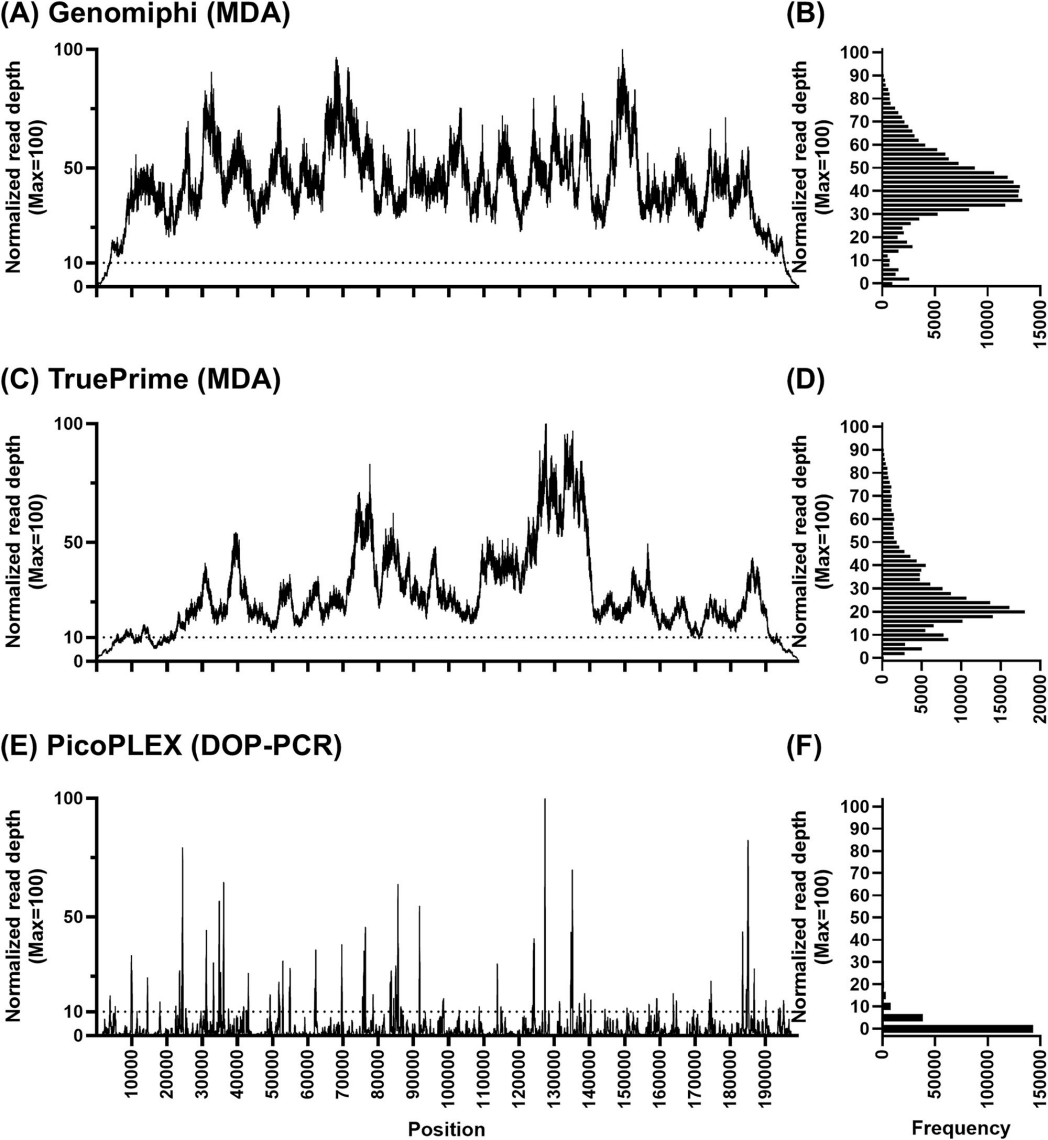
Choice of WGA method influences read distribution across the MPXV genome. NGS reads from MPXV strain Sierra Leone enriched using different WGA methods were aligned to the reference genome. **A**, **C**, **E** Read depth distribution using GenomiPhi, TruePrime, and PicoPLEX, respectively. Read depths are normalized to a maximum of 100. The corresponding histograms of the normalized read depth frequency are shown in (**B**, **D**, **F**).

Nucleic acids were purified from an MPXV Sierra Leone working stock following micrococcal nuclease treatment. The DNA was then amplified using three WGA methods: GenomiPhi V3, which uses random hexamer primers and Phi29 polymerase for isothermal amplification; TruePrime, which also uses Phi29 polymerase but synthesizes primers in situ via a primase/polymerase complex^19^; and PicoPLEX, which uses degenerate primers and PCR-based thermal cycling to amplify whole genomes from minimal DNA input^20^. The amplified DNA was used for library preparation using an Illumina kit, and the libraries were sequenced on the Illumina iSeq100 platform. The sequencing reads were aligned to the MPXV Sierra Leone reference genome, and relative read depth was normalized to a maximum of 100 (Fig. 4A).

GenomiPhi provided the most unbiased coverage across the viral genome among the three methods tested (Fig. 4A, C, E). Furthermore, the GenomiPhi-prepared library exhibited the highest median relative read depth frequency (Fig. 4B, D, F). These findings indicated that GenomiPhi is optimal for unbiased poxvirus WGA and that the choice of WGA method significantly affects NGS results.

### Terminal PCR compensates for reduced read depth at genome termini in MPXV due to MDA

While nuclease–MDA effectively enriches the MPXV genome, coverage at genome termini is often reduced by MDA, as shown in Fig. 4A. This limitation can be compensated for by terminal PCR, which uses primers targeting highly conserved sequences to amplify ~8 kb at the 5′ end and ~9 kb at the 3′ end, generating PCR products that reliably cover the terminal regions (Supplementary Fig. 1A).

We assessed the effect of terminal PCR using MPXV working stocks, with or without micrococcal nuclease treatment. The 5′ and 3′ ends were amplified separately, using the primers listed in Supplementary Table 1. As shown in Supplementary Fig. 1B and C, terminal genome regions in both clade I and clade II strains were efficiently amplified. Purified amplicons were used for NGS library preparation and sequencing.

### Sensitivity and sequencing efficiency of the nuclease–MDA method in the presence of host DNA

To evaluate our method for clinical application, we determined its Limit of Detection (LOD). A 10-fold serial dilution series of the MPXV strain Congo-8 was prepared in media containing host genomic DNA from the freeze-thaw lysate of uninfected, confluent RK13 cells. This experimental design mimics low-titer clinical samples that are heavily contaminated with host DNA, which is a common challenge in routine surveillance.

The nuclease–MDA method followed by MinION sequencing achieved >90% genome coverage at 10× depth down to an input of 40 Plaque Forming Units (PFU) (corresponding to a Ct value of 33.5) (Table 1). In contrast, an input of 4 PFU (corresponding to a Ct value of 37.3) yielded an impractically large number of MPXV-derived reads. This demonstrates that the practical LOD of our assay is approximately 40 PFU (referencing the original viral titer contained in the 10 µL purified nucleic acid aliquot used for the MDA reaction).

**Table 1:** Evaluation of the limit of detection and sequencing efficiency of the nuclease–MDA method in the presence of host DNA background

MPXV titer (PFU)^a^	Ct value^b^	Total reads	Mapped reads	% mapped reads in total reads	Median depth	>10× coverage rate (%)	>50× coverage rate (%)
1260	26.0	52,280	33,629	64.3	174	99.9	96.5
94	30.3	34,218	22,410	65.5	117	99.4	92.7
14.2	33.5	45,503	9776	21.5	35	90.0	33.2
0.4	37.3	35,711	200	0.6	0	0.1	0.0

^a^MPXV titer represents the original viral infectivity units contained in the 40 μL virus suspension, which was the source of the 10 μL purified nucleic acid used for the MDA reaction. This value is the actual measured back-titer derived from the 10-fold serially diluted virus solution used in the experiment.

^b^The Ct value was derived from qPCR targeting the MPXV gene using the purified nucleic acid.

These experiments demonstrated that the nuclease–MDA method has sufficient sensitivity and enrichment efficiency to obtain near-complete MPXV whole-genome sequences from clinical-like samples with both low viral loads and high host DNA contamination.

### NGS performance after nuclease–MDA treatment and terminal PCR

The nuclease–MDA method was used to determine the whole-genome sequences of 18 MPXV strains (Supplementary Table 2) stored at the NIID, using the MinION platform. The sequencing reads were mapped to the reference genome using minimap2. To enhance accuracy, the reference genome used for mapping incorporated terminal sequences determined using a library enriched by terminal PCR in conjunction with the nuclease–MDA method. The genome sequences have been deposited in the DDBJ/EMBL/NCBI GenBank database as shown in Supplementary Table 2.

Table 2 summarizes the NGS run statistics for each strain, sorted in ascending order of total read count to assess the relationship between total reads and genome-wide read depth distribution. Across all sequenced strains, more than 96% of total mapped reads were specific to MPXV, demonstrating the consistent enrichment efficiency of this method.

**Table 2:** Summary of NGS results for MPXV strains enriched by nuclease–MDA

Strain	Clade	Genome size (bp)	Total reads	Mapped reads	% mapped reads in total reads
Congo-8	Ia	196,860	71,064	70,650	99.4
V96-I-003	Ia	196,935	16,040	15,999	99.7
V96-I-004	Ia	196,918	16,033	15,831	98.7
V96-I-005	Ia	196,940	12,011	11,987	99.8
V96-I-008	Ia	196,940	20,116	19,926	99.1
V96-I-016	Ia	196,942	20,042	20,009	99.8
V96-I-017	Ia	196,963	8040	8010	99.6
V96-I-062B	Ia	197,076	16,032	16,013	99.9
V96-I-063B	Ia	197,080	20,049	20,016	99.8
V96-I-071	Ia	197,087	26,045	25,736	98.8
V96-I-079A	Ia	196,943	16,032	16,015	99.9
Zr-599	Ia	196,793	94,783	91,549	96.6
Anteater	IIa	199,429	19,305	19,229	99.6
Copenhagen	IIa	199,642	21,269	21,113	99.3
Liberia	IIa	199,049	51,547	49,653	96.3
Orangutan	IIa	199,298	25,634	25,521	99.6
SEN-70	IIa	199,250	221,307	220,975	99.8
SierraLeone	IIa	198,946	130,397	130,249	99.9

**Fig. 5:**
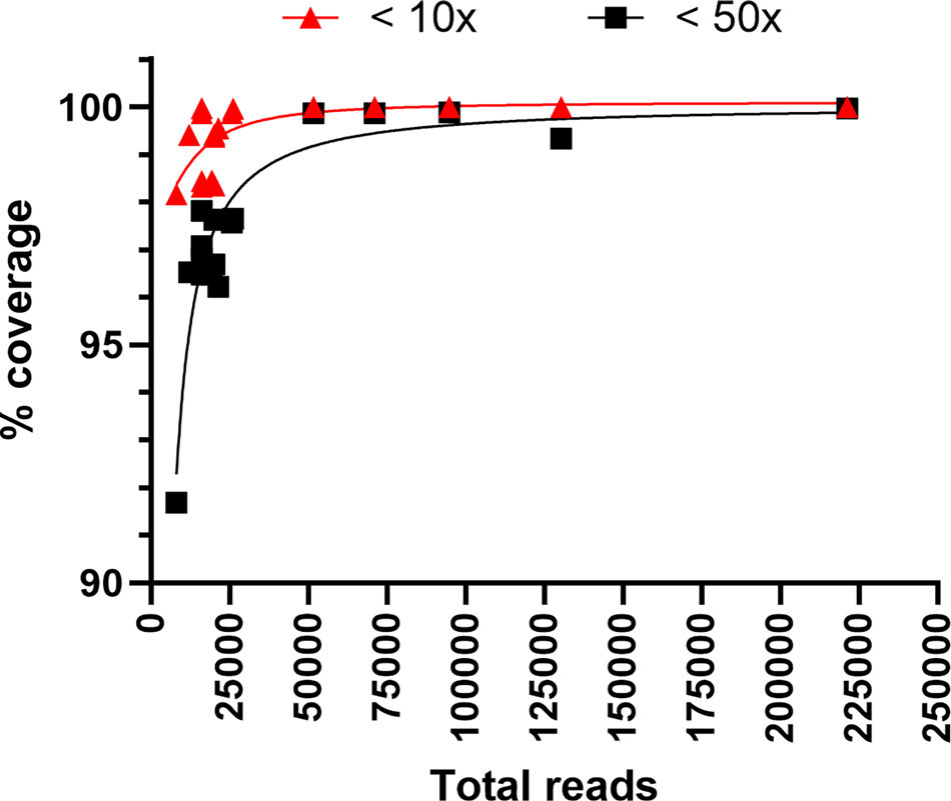
Relationship between total reads and percent of coverage at 10× or 50× depth in NGS runs. For each strain listed in Tables 3 and 4, the total number of reads was plotted against the percentage of coverage in the genome at >10× (red squares) or >50× (black squares) after mapping to the reference genome. Four-parameter logistic curves were fitted to each of the dataset.

As the number of total reads increased, the median read depth and the percent of coverage in the genome at 10× or 50× depth also increased (Table 3, Fig. 5). The percent of coverage at 10× or 50× sharply increased until that of total reads reached ~25,000 (Fig. 5). Beyond 50,000 total reads, read depth distribution stabilized, with no genomic regions falling below 10× read depth, and the total length of regions with read depth below 50× was reduced to fewer than 1300 bases. These results suggested that obtaining at least 10,000 total reads is sufficient for generating a draft whole-genome sequence, whereas 50,000 or more reads ensure that the entire genome achieves a minimum read depth of 10×, facilitating more reliable genome assembly with improved completeness and accuracy. Terminal PCR can be employed to improve the accuracy of sequence determination at genome termini, where read depth tends to be reduced.

**Table 3:** Relationship between total reads and genomic regions achieving 10× and 50× read depth

Strain	Total reads	Median depth (×)	>10× position (5′–3′)	>10× coverage rate (%)	>50× position (5′–3′)	>50× coverage rate (%)
SEN-70	221,307	1723	1-end	100.00	27-199224	99.97
Sierra Leone	130,397	1042	1-end	100.00	651-198278	99.33
Zr-599	94,783	1234	1-end	100.00	130-196664	99.88
Congo-8	71,064	925	1-end	100.00	130-196731	99.87
Liberia	51,547	634	1-end	100.00	131-198919	99.87
V96-I-071	26,045	313	40-197048	99.96	2315-194770	97.65
Orangutan	25,634	328	131-199168	99.87	2233-196748	97.57
Copenhagen	21,269	248	444-199199	99.55	3755-195953	96.22
V96-I-008	20,116	179	1623-195318	98.35	3284-193709	96.69
V96-I-063B	20,049	202	620-196461	99.37	2345-194736	97.62
V96-I-016	20,042	192	568-196375	99.42	2333-194610	97.63
Anteater	19,305	246	1522-197908	98.45	3510-196008	96.48
V96-I-003	16,040	155	1656-195281	98.34	3448-193447	96.52
V96-I-004	16,033	163	131-196788	99.87	2174-194774	97.84
V96-I-062B	16,032	153	26-197051	99.97	2922-194155	97.06
V96-I-079A	16,032	159	1532-195412	98.46	3138-193813	96.85
V96-I-005	12,011	117	585-196356	99.41	3559-193642	96.56
V96-I-017	8040	119	1638-194983	98.18	6909-187521	91.78

### Molecular phylogenetic analysis

We conducted a molecular phylogenetic analysis to determine the phylogenetic characteristics of each strain and to assess whether the NIID strains were closely related to previously deposited strains by other groups (Supplementary Table 4).

**Fig. 6:**
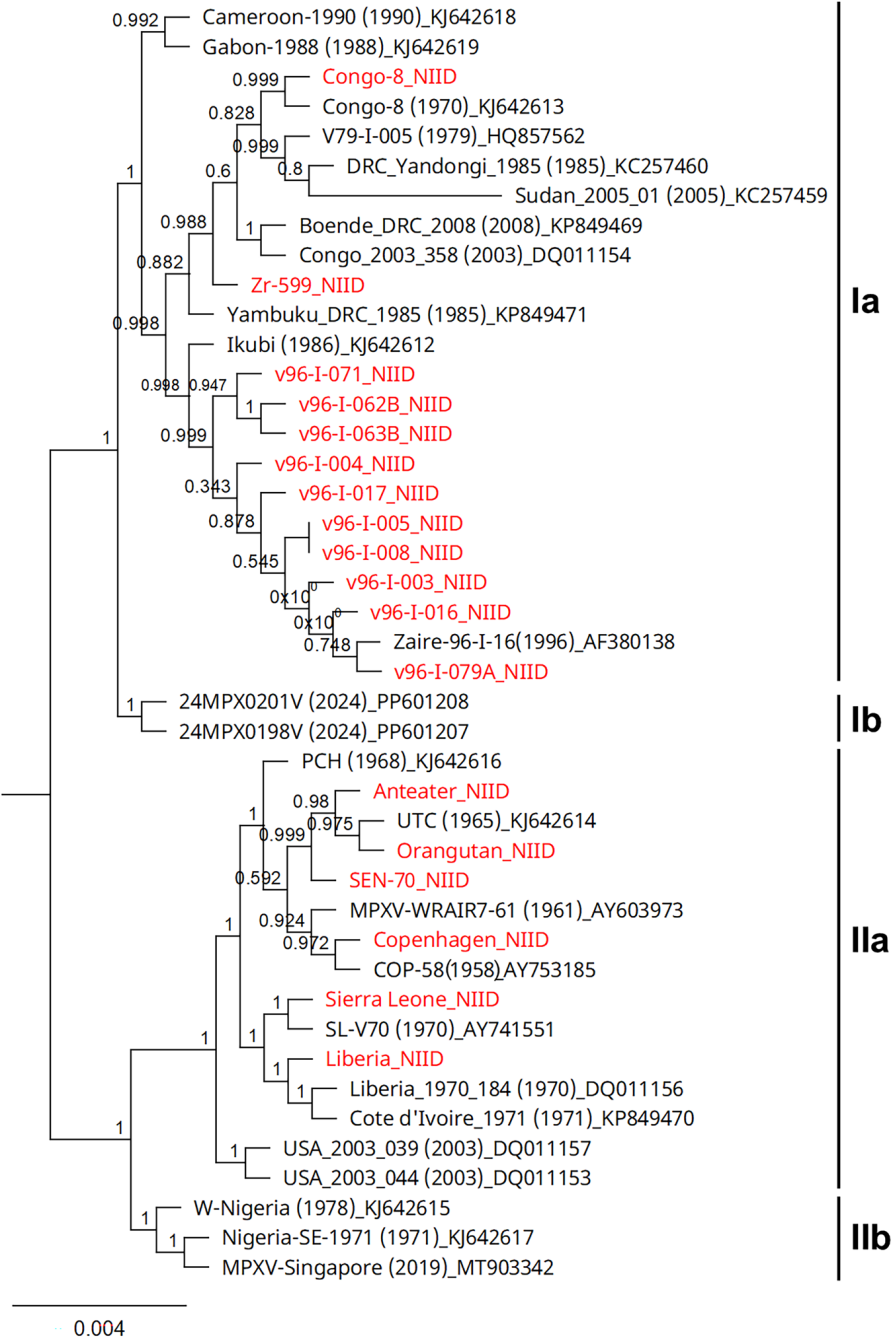
Phylogenetic analysis of MPXV strains. Phylogenetic analysis was performed using whole-genome sequences of MPXV strains stored at the NIID (red) and publicly available strains (black). An approximately maximum-likelihood phylogenetic tree was constructed using FastTree v2.1.12. Years of isolation are indicated in parentheses, with accession numbers appended to strain names. The tree was generated using FastTree2. The vaccinia virus strain Copenhagen (accession No. OP868847) was used as the outgroup but is not shown. Branch lengths are to scale. Support values from FastTree are shown at nodes to indicate branch reliability.

As shown in Fig. 6, the 18 NIID strains were distributed within the clade Ia and IIa lineages. Genetic comparisons, considering relevant literature, revealed that certain strains were genetically identical, with only minor differences, particularly in terms of homopolymer stretches (e.g., poly-A length) and tandem repeat copy numbers, likely because of variations in passage history and sequencing accuracy. Specifically, the two Congo-8 strains, Sierra Leone and SL-V70, Liberia and Liberia_1970_184, and Copenhagen and COP-58 were identified as identical strains.

The V96-I lineage strains formed a distinct cluster with Zaire-96-I-016, which was isolated during the 1996 outbreak in Zaire^21^, supporting the consistency of their classification. The distribution of single-nucleotide polymorphisms and tandem repeat variations across the V96-I genomes strongly suggests that these strains were derived from multiple patients infected during a single outbreak event, likely stemming from a common source.

The Anteater and Orangutan MPXV strains, clustered closely with the UTC strain, which were isolated during the 1965 Rotterdam Zoo outbreak^22^.

### Applicability of the nuclease–MDA method to other poxviruses

To evaluate whether the established nuclease–MDA method is applicable to other poxviruses besides MPXV, we sequenced four strains using the same enrichment protocol: three strains of Cowpox virus (CPXV: Amsterdam, Brighton Red, and Strain 58) and one strain of Ectromelia virus (ECTV: Hampstead) (Supplementary Table 5). The genome sequences have been deposited in the DDBJ/EMBL/NCBI GenBank database as shown in Supplementary Table 5.

The sequencing results for these strains, summarized in Table 4, demonstrated favorable performance comparable to that obtained for MPXV strains (Table 3). Specifically, the proportion of Poxvirus-specific reads among all reads was consistently high (72.0–89.4%), indicating highly efficient genome enrichment and successful host DNA depletion.

**Table 4:** Summary of the NGS results for CPXV and ECTV strains enriched by nuclease–MDA

Virus	Strain	Working stock titer (PFU/ml)	Total reads	Mapped reads	% mapped reads in total reads	Median depth	>10x coverage rate (%)	>50x coverage rate (%)
CPXV	58	1.0 × 10^6^	60,459	51,375	85.0	232	100	97.9
CPXV	Amsterdam	2.5 × 10^4^	42,216	30,381	72.0	153	99.8	97.1
CPXV	Brighton Red	2.6 × 10^4^	36,979	33,076	89.4	182	99.4	98.5
ECTV	Hampstead	1.2 × 10^6^	24,311	20,284	83.4	112	100	94.1

Consequently, the percent coverage at a depth of 10x was above 99% for all four strains, and the coverage at 50x was also high (94.1–97.9%). These results enabled the successful whole-genome assembly of all four CPXV and ECTV strains.

Collectively, these findings demonstrate that the nuclease–MDA method is a versatile and robust strategy suitable for efficient WGS across various Poxvirus strains, not limited to MPXV.

## Discussion

This study developed a novel enrichment strategy to overcome the technical challenges in MPXV WGS, namely, the high host DNA burden, large genome size, and limitations of conventional methods. We established the nuclease–MDA method, which combines selective host DNA degradation via Micrococcal nuclease treatment with MDA using GenomiPhi. This combination achieves a synergistic enrichment of viral DNA. Furthermore, we optimized the method by adding Terminal PCR to compensate for the reduced read depth typically observed at the genome termini. Application of this integrated strategy to 18 MPXV isolates demonstrated high efficiency and robustness (Table 3), yielding an average of over 96% MPXV-specific reads and enabling complete genome assembly for all strains. We confirmed that using GenomiPhi for MDA was essential for obtaining high-quality sequence data because of its superior read depth uniformity (Fig. 4).

Phylogenetic analysis based on whole-genome sequences of MPXV isolates from the 1996–1997 outbreak revealed that these strains formed a single cluster and were closely related (Fig. 6). This finding is consistent with contemporaneous epidemiological observations, which indicate sustained human-to-human transmission during the outbreak^23^. The genomic similarity and coherent phylogenetic placement of the V96-I lineage strains, including those newly sequenced in the present study, strongly suggest that this outbreak originated from a single zoonotic spillover event, followed by human-to-human transmission involving multiple patients. The UTC strain clustered tightly with the anteater and orangutan strains (Fig. 6). This outbreak is considered to have been triggered by the introduction of an anteater infected with a clade IIa MPXV, for reasons that remain unclear, which subsequently transmitted the virus to other captive animals^22^. Our phylogenetic findings support this interpretation.

We quantitatively evaluated the sensitivity of our method by determining its Limit of Detection (LoD). Titration experiments demonstrated that our approach achieved sufficient WGS coverage even from a low viral DNA copy number corresponding to a Ct value of 33.5. This suggests a performance level possibly comparable to that of reported PCR-based enrichment methods, which typically show a sharp 10x coverage rate decline above Ct 30 (e.g., from ~80 to ~40% between Ct 32 and 34)^16^. This low LoD is attributable to the synergistic effect of efficient host DNA removal and MDA’s high amplification capacity. Given its practicality relative to clinical viral loads, our method suggests the potential for direct WGS from unprocessed clinical samples, thereby enhancing the speed and utility of genomic surveillance.

Our nuclease–MDA method offers a competitive and viable alternative to other MPXV genome enrichment techniques (Table 5). Notably, the primer-free nature of MDA entirely avoids the risk of coverage dropout associated with primer mismatches in PCR-based enrichment. Moreover, it offers superior cost efficiency compared to the expensive Hybridization Capture method. The broader applicability of this method to other poxviruses also distinguishes it from the sequence-specific Specific-octamer MDA approach, which remains dependent on MPXV-specific primers. Finally, our technique is practical for low-titer samples (Ct > 30) likely due to the MDA amplification step, distinguishing it from the Saponin and Nuclease-Pretreated Metagenomics approach, which is typically restricted to samples with Ct values below 30^18^.

**Table 5:** Comparison of MPXV genome enrichment methods

Enrichment method	Core principle	Advantages	Limitations	References
Hybridization capture	Sequence-specific probe capture	- Directly applicable to clinical specimens - Maintains high read depth across broad regions even at low viral copy numbers	- High cost of custom probe synthesis - Risk of enrichment failure because of probe mismatches due to viral mutations	^15^
PCR-based enrichment	Multiplex tiling PCR	- Directly applicable to clinical specimens - Cost-efficient - Enables sensitive detection of specific genomic regions	- Read depth is limited to fewer regions at low viral copy number - Risk of amplification failure due to primer mismatches	^7,16^
Specific-octamer MDA	Specific-primer (octamer) MDA	- Directly applicable to clinical specimen - Low risk of amplification failure due to primer mismatches	- Reduced read depth at genome termini	^17^
Saponin and nuclease-pretreated metagenomics	Host DNA degradation and concentration (no amplification)	- Directly applicable to clinical specimens	- Inefficient for low-titer samples (Ct > 30)	^18^
Nuclease–MDA with terminal PCR	Host DNA degradation and random-primer MDA	- Cost-efficient - Enables primer-free WGA - Adaptable to other poxviruses and dsDNA viruses	- Reduced read depth at genome termini (Terminal PCR compensates) - Lacks validation on clinical samples	This study

The high efficiency of the nuclease–MDA method extends to other Poxvirus strains, including CPXV and ECTV (Table 4). This versatility is likely due to the high compatibility between the characteristic large linear dsDNA genome of poxviruses and the MDA amplification mechanism (random priming and strand displacement). Based on this principle, we propose that this method also holds potential for the WGS of other dsDNA viruses beyond the *Poxviridae*, such as Herpesviruses, warranting further investigation.

In conclusion, the nuclease–MDA method provides a highly efficient, cost-effective, and robust WGS platform for MPXV and other poxviruses. This technique represents a valuable tool for rapid phylogenetic surveillance and fundamental research across the *Poxviridae*.

## Methods

### Cells and viruses

Rabbit kidney-derived RK-13 cells (maintained at the NIID since at least the 1990s), along with Vero cells (CCL-81, ATCC, Manassas, VA), Vero E6 cells (CRL-1586, ATCC), and L929 cells (IFO50409, JCRB cell bank, Osaka, Japan), were cultured in Dulbecco’s modified Eagle’s medium (catalog No. 041-30081; Wako, Osaka, Japan) supplemented with 5% heat-inactivated fetal bovine serum, 100 U/mL penicillin, and 100 μg/mL streptomycin (catalog No. 15140122; Thermo Fisher Scientific, Waltham, MA). When necessary, BIOMYC-3 (Mycoplasma spp. antibiotic; catalog No. PK-CC03-038-1D; Takara Bio, Shiga, Japan) was added at a 100-fold dilution.

Eighteen MPXV strains, three CPXV strains, one ECTV strain, and m8 (GenBank Accession no. AB687720), were prepared from samples kindly provided by various researchers in the past and preserved at the NIID as chorioallantoic membrane (CAM) or cell culture supernatant samples at –80 °C. Details of all MPXV, CPXV, and ECTV strains are presented in Supplementary Table 2 and Supplementary Table 5. All viruses were propagated starting from the preserved samples: MPXV strains were initially passaged in VeroE6 cells and then propagated in RK-13 cells; m8, which was gifted by Dr. So Hashizume (Chiba Serum Institute) was propagated in RK-13 cells; CPXV strains were propagated in Vero cells; and the ECTV strain was propagated in L929 cells. All viruses were used for four or fewer passages. Viruses were grown at a multiplicity of infection of 0.01–0.1, and cells and culture supernatants were harvested upon full cytopathic effect, subjected to a single freeze-thaw cycle to release cell-associated viruses, and centrifuged at 1000 × *g* for 10 min. The supernatants were used as the working stocks. The stocks were aliquoted and stored at –80 °C, and the infectious titers of all working stocks (MPXV: 1 × 10^6^ to 2 × 10^7^ PFU/mL; CPXV, ECTV, and m8) were determined using a standard plaque assay in 24-well plates.

### Nucleic acid purification for poxviral genomic DNA enrichment

Step-by-step protocols for the nuclease–MDA and terminal PCR methods are available as in the protocols.io repository^24,25^.

The virus working stock was centrifuged at 6000 × *g* for 10 min to remove cellular debris. The supernatant was treated with the non-specific endo-exonuclease Micrococcal Nuclease (catalog No. M0247S; NEB, Ipswich, MA) to digest unwanted nucleic acids while preserving the viral DNA. The 200-μL reaction mixture, including 179 μL of supernatant, 1 μL of Micrococcal Nuclease, and 20 μL of 10× buffer, were incubated at 37 °C for 1 h. For comparative experiments, Micrococcal Nuclease and its buffer were replaced with OmniCleave Endonuclease (catalog No. OC7850K; LGC Biosearch Technologies, UK) or RQ1 RNase-Free DNase (catalog No. M6101; Promega, Madison, WI).

Following nuclease treatment, poxviral genomic DNA was purified using the High Pure Viral Nucleic Acid Kit (catalog No. 11858874001; Roche Applied Science, Penzberg, Germany) per the manufacturer’s instructions. The final elution volume was 50 μL.

### Nucleic acid amplification by MDA for poxviral genomic DNA enrichment

Poxviral DNA was amplified using the Illustra Ready-To-Go GenomiPhi V3 DNA Amplification Kit (catalog No. 25-6601-24; Cytiva, Tokyo, Japan) according to the manufacturer’s instructions, except that the reaction time was extended from 1.5 to 4 h. The DNA solution (10 μL) was mixed with 2× denaturation buffer (10 μL), incubated at 95 °C for 3 min, and cooled to 4 °C. A Ready-To-Go GenomiPhi cake was added to the reaction mixture (final volume: 20 μL), followed by incubation at 30 °C for 4 h and 65 °C for 10 min.

The amplified DNA was purified using AMPure XP reagent (0.5-fold the volume), washed twice with 100 μL of 70% ethanol, and eluted with 40 μL of H_2_O after incubation at 37 °C for 10 min. The total DNA yield was measured using a Qubit 4 Fluorometer and the Qubit 1X dsDNA BR Assay Kit (Thermo Fisher Scientific). Comparative experiments were conducted using the 4BB TruePrime WGA Kit (catalog No. 370025; 4basebio, Heidelberg, Germany) and PicoPLEX WGA Kit (catalog No. R30050; Takara Bio) following the respective manufacturer’s protocols.

### Terminal PCR to amplify 5′ and 3′ poxviral termini

The 5**′** and 3′ termini of the MPXV genome were PCR-amplified using DNA extracted with the High Pure Viral Nucleic Acid Kit. Primers (Supplementary Table 1) were designed using DNAdynamo based on the MPV-ZAI reference sequence (GenBank Accession No. AF380138) reported by Shchelkunov et al.^26^. PCR produced ~8-kb and ~9-kb amplicons for the 5′ and 3′ ends, respectively.

Each 20-μL PCR mixture contained 1 μL of DNA, 10 μL of KOD One PCR Master Mix (catalog No. KMM-101; Toyobo, Osaka, Japan), 1 μL of a 5-μM primer mix (0.25 μM final concentration per primer), and 7 μL of H_2_O. The thermal cycling conditions included initial denaturation at 98 °C for 15 s, followed by five cycles of 98 °C for 10 s, 65 °C for 5 s, and 68 °C for 90 s with a 1 °C decrement per cycle, then 35 cycles of 98 °C for 10 s, 60 °C for 5 s, and 68 °C for 90 s.

The PCR products were purified using AMPure XP reagent (0.5-fold volume), washed twice with 100 μL of 70% ethanol, and eluted in 20 μL of H_2_O after incubation at 37 °C for 10 min. DNA concentrations were determined using a Qubit 4 Fluorometer and the Qubit 1X dsDNA BR Assay Kit.

### Library preparation and MinION sequencing

Libraries were prepared using a protocol based on the ONT Ligation Sequencing gDNA V14 – Whole Genome Amplification (SQK-LSK114, WAL_9192_v114_revD_26Jul2023) and the Native Barcoding Kit 24 V14 (SQK-NBD114.24, NBE_9169_v114_revQ_15Sep2022), with minor modifications.

GenomiPhi-amplified DNA was first treated with T7 Endonuclease I (catalog No. M0302S; NEB) to remove branched structures. The 30-μL reaction mixture, including 1 μg DNA, 3 μL of NEBuffer 2, 1.5 μL of T7 Endonuclease I, and nuclease-free water, was incubated at 37 °C for 30 min, yielding a median library fragment length of approximately 2000 bp. The DNA was purified using AMPure XP Reagent (0.5× volume), washed twice with 100 μL of 70% ethanol, and eluted with 13 μL of nuclease-free water.

Subsequently, DNA repair and end-preparation were performed using the NEBNext FFPE DNA Repair Mix (catalog No. M6630; NEB) and NEBNext Ultra II End Repair/dA-Tailing Module (catalog No. E7546; NEB). The 15-μL reaction mixture, including 12 μL of DNA, 0.5 μL of FFPE DNA Repair Mix, 0.875 μL of FFPE DNA Repair Buffer, 0.75 μL of End-Prep Enzyme Mix, and 0.875 μL of End-Prep Reaction Buffer was incubated at 20 °C for 30 min, followed by 65 °C for 5 min. The repaired DNA was purified with AMPure XP Reagent (1.0× volume), washed twice with 100 μL of 70% ethanol, and eluted in 10 μL of H_2_O.

For samples amplified using terminal PCR, end-preparation of purified PCR products was performed using the NEBNext Ultra II End Repair/dA-Tailing Module (NEB) in a 15-μL reaction containing up to 200 fmol of DNA (e.g., 1100 ng for 9-kb amplicons) in 12.5 μL, along with 1.75 μL of Ultra II End-Prep Reaction Buffer and 0.75 μL of Ultra II End-Prep Enzyme Mix. The reaction was incubated at 20 °C for 5 min, then at 65°C for 5 min. The DNA was purified using AMPure XP Reagent (0.5-fold the volume), washed twice with 100 μL of 70% ethanol, and eluted with 10 μL of nuclease-free water after 37 °C incubation for 10 min.

For barcoding, DNA was ligated using a Native Barcoding Kit 24 or 96 V14 (SQK-NBD114.24 or 114.96, ONT) in a 20-μL reaction mixture including 8.5 μL of DNA, 10 μL of Blunt/TA Ligase Master Mix (catalog No. M0367S; NEB), and 1.5 μL of Native Barcoding Expansion. The reaction was incubated at room temperature (20–25 °C) for 20 min and then mixed with 2 μL of EDTA. Then, the DNA samples were pooled, purified with AMPure XP Reagent (0.4-fold volume), washed twice with 700 μL of 70% ethanol, and eluted in 35 μL (for pools of ≥9 samples), 0.2-fold volume (for pools of 3–9 samples), or 12 μL of H_2_O (for pools of <3 samples).

Sequencing adapters were ligated in a 20-μL reaction mixture consisting of 12 μL of barcoded DNA, 2 μL of Native Adapter (NA), 4 μL of 5× NEBNext Quick Ligation Buffer, and 2 μL of Quick T4 DNA Ligase (catalog No. E6056S; NEB). The mixture was incubated at room temperature for 20 min. Adapter-ligated DNA was purified using 10 μL of AMPure XP Reagent, washed twice with 100 μL of Short Fragment Buffer, and eluted with 15 μL of Elution Buffer provided in the sequencing kit.

The DNA concentration in the final library was measured using a Qubit 4 Fluorometer and Qubit 1X dsDNA BR Assay Kit. The molar concentration was calculated based on an estimated median library fragment length of ~2000 bp, as previously reported^27^. The DNA (10–20 fmol) was loaded onto a MinION flow cell for sequencing, according to the manufacturer’s protocol. The raw data were basecalled in the super-accurate mode during the NGS run, using the MinKNOW control software. Demultiplexing and barcode trimming of the barcoded samples were performed using the MinKNOW control software.

In some cases, the Rapid Barcoding Kit 96 V14 (SQK-RBK114.96, ONT) was used instead of the Native Barcoding Kit (SQK-NBD114.24 or 114.96). 100 ng of the GenomiPhi-amplified DNA was directly used as input for the barcoding step of the manufacturer’s protocol (ONT document RBK_9176_v114_revS_27Oct2025), omitting the DNA repair and end-preparation steps (including T7 Endonuclease I treatment) given the kit’s transposase-based cleavage and barcoding.

### Illumina iSeq 100 sequencing

Libraries were prepared using Illumina DNA Prep, (M) Tagmentation (catalog No. 20060060; Illumina, San Diego, CA) following the Illumina DNA Prep Reference Guide (document No. 1000000025416 v10, August 2021).

Tagmentation was conducted using 500 ng of DNA (amplified with GenomiPhi V3 or other WGA methods) in 30 µL including 10 µL of Bead-Linked Transposomes and 10 µL of Tagment Buffer 1. The reaction mixture was incubated at 55 °C for 15 min. For post-tagmentation cleanup, 10 µL of Tagment Stop Buffer was added, the beads were resuspended, and the mixture was incubated at 37 °C for 15 min. The reaction was placed on a magnetic stand, and the beads were washed twice with 100 µL of Tagment Wash Buffer.

For PCR amplification, 40 µL of Enhancement PCR Mix was added directly to the beads, followed by 10 µL of an indexed adapter (e.g., IDT for Illumina DNA/RNA UD Indexes Set A, catalog No. 20027213). The thermal cycles were as follows: 68 °C for 3 min, 98 °C for 3 min, and 5 cycles of 98 °C for 45 s, 62 °C for 30 s, and 68 °C for 1 min.

The libraries were cleaned up using Illumina Purification Beads and quantified using a Qubit 4 Fluorometer and Qubit 1X dsDNA BR Assay Kit. The molar concentration was calculated assuming an average library size of 600 bases. Twenty microliters of a pooled library (20 pM) was loaded onto the Illumina iSeq100 system for paired-end sequencing with a read length of 150 nucleotides, according to the manufacturer’s protocol.

Demultiplexing and barcode trimming of the barcoded samples were performed using the Generate FASTQ module on the Local Run Manager built into the iSeq100 system. Sample-specific FASTQ files obtained after the NGS run completion were used for subsequent analysis.

### Sequence alignment

The sample-specific FASTQ files obtained after the NGS run completion were used for subsequent analysis. The reads in the FASTQ files were aligned to the reference viral genome, initially using strain Zaire-96-I-16 (NCBI accession number: AF380138) or Liberia_1970_184 (NCBI accession number: DQ011156) using the Map to Reference function in Geneious Prime version 2025.0.3 (Dotmatics, Boston, MA). Alignment was performed using the Minimap2^28^ plugin (version 2.24). The alignment was run with Minimap2’s default settings, except that the data type was set to Oxford Nanopore or Short Reads, depending on the data source. Key settings included: maximum secondary alignments per read was 5, and the minimum secondary to primary alignment score ratio was 0.8. Reads were not trimmed before mapping. The resulting assembled sequence was saved as the initial draft genome, which was then used as a reference for at least three rounds of polishing to determine the complete genome sequence.

### qPCR

Genome copies of m8 and RK-13 cells in purified or amplified nucleic acid solutions were quantified using qPCR targeting the *G9R* gene of m8 and the rabbit *GAPDH* gene, respectively (Supplementary Table 3). Two microliters of the sample was added to a 10 μl reaction mixture containing 2X QuantiTect SYBR Green PCR Master Mix (QuantiTect SYBR Green PCR Kit, catalog No. 204143; Qiagen, Hilden, Germany), 0.4 μM of each primer, and H_2_O. PCRs were run in a LightCycler Nano instrument (Roche), with an initial denaturation at 95 °C for 15 min, followed by 45 cycles of 95 °C for 15 s and 60°C for 1 min.

MPXV genome copies in purified nucleic acid solutions were quantified using qPCR targeting the F3L gene. This protocol was a modification of the method previously reported by Maksyutov et al.^29^. Specifically, the fluorescent dyes of the hydrolysis probes were modified: the dye for the MPXV-specific probe was changed from JOE to HEX, and the dye for the VZV-specific probe was changed from TAMRA to Texas Red. The qPCR reaction mixture was prepared by adding 2 μl of sample to a 10 μl reaction mixture containing 2X QuantiTect Probe PCR Master Mix (QuantiTect Probe PCR Kit, catalog No. 204343), 0.2 μM of each primer and probe, and H_2_O. PCRs were performed using a LightCycler 96 instrument (Roche). The thermocycling conditions consisted of an initial denaturation at 95 °C for 10 min, followed by 45 cycles of 95 °C for 15 s and 60°C for 1 min.

### Phylogenetic analysis

Phylogenetic analysis was performed using the 18 whole-genome sequences generated in this study, along with MPXV whole-genome sequences available in the DDBJ/EMBL/NCBI GenBank databases (Supplementary Table 4).

The MPXV genomes were aligned using MAFFT v7.490^30^. The MAFFT plugin in Geneious Prime 2025.0.3 was run using the default settings: Algorithm set to ‘Auto’, Scoring matrix set to ‘200PAM/k = 2’, Gap open penalty set to 1.53, and Offset value set to 0.123.

An approximately maximum-likelihood phylogenetic tree was constructed using FastTree v2.1.12^31,32^, a program designed to estimate phylogenies from large multiple sequence alignments. The FastTree plugin in Geneious Prime 2025.0.3 was run using the software’s default settings: none of the options were checked, the number of “Rate categories of sites” was set to 20, and the default Jukes-Cantor model was used (the “Use Generalized Time-Reversible (GTR) rather than Jukes-Cantor model” box was unchecked). The vaccinia virus strain Copenhagen (accession No. OP868847) was used as an outgroup taxon.

### Statistical analysis

All statistical analyses were conducted using GraphPad Prism 10 (GraphPad Software, La Jolla, CA). The statistical methods used for each experiment are described in the figure legends. Statistical significance was set to *p* < 0.05.

## Supplementary information

Supplementary Information

## Data Availability

The NGS data for all strains generated in this study have been deposited in the DDBJ/EMBL/NCBI Sequence Read Archive (SRA) under BioProject accession number PRJNA1312998. The complete whole-genome sequences of all Monkeypox Virus (MPXV), Cowpox Virus (CPXV), and Ectromelia Virus (ECTV) strains determined in this study have been deposited in the GenBank database. The MPXV strains deposited are: Congo-8 (PX119012), V96-I-003 (PX119018), V96-I-004 (PX119019), V96-I-005 (PX119020), V96-I-008 (PX119021), V96-I-016 (PX119022), V96-I-017 (PX119023), V96-I-062B (PX119024), V96-I-063B (PX119025), V96-I-071 (PX119026), V96-I-079A (PX119027), Zr-599 (PX119028), Anteater (PX122227), Copenhagen (PX119013), Liberia (PX119014), Orangutan (PX119015), SEN-70 (PX119016), and Sierra Leone (PX119017). The CPXV strains deposited are: 58 (PX585980), Amsterdam (PX585981), and Brighton Red (PX585982). The ECTV strain deposited is: Hampstead (PX585983). The corresponding accession numbers for all sequenced strains are also summarized in Supplementary Tables 2 and 5.

## References

[1] Ladnyj, I. D., Ziegler, P. & Kima, E. A human infection caused by monkeypox virus in Basankusu Territory, Democratic Republic of the Congo. *Bull. World Health Organ* **46**, 593–597 (1972). 4340218 PMC2480792

[2] Brown, K. & Leggat, P. A. Human monkeypox: current state of knowledge and implications for the future. *Trop. Med. Infect. Dis.* **1**, 10.3390/tropicalmed1010008 (2016). 10.3390/tropicalmed1010008 PMC6082047 30270859

[3] Bunge, E. M. et al. The changing epidemiology of human monkeypox-A potential threat? A systematic review. *PLoS Negl. Trop. Dis.* **16**, e0010141 (2022). 35148313 10.1371/journal.pntd.0010141 PMC8870502

[4] Tatem, A. J., Rogers, D. J. & Hay, S. I. Global transport networks and infectious disease spread. *Adv. Parasitol.* **62**, 293–343 (2006). 16647974 10.1016/S0065-308X(05)62009-X PMC3145127

[5] Minhaj, F. S. et al. Monkeypox Outbreak - Nine States, May 2022. *MMWR Morb. Mortal. Wkly Rep.* **71**, 764–769 (2022). 35679181 10.15585/mmwr.mm7123e1 PMC9181052

[6] Thornhill, J. P. et al. Monkeypox virus infection in humans across 16 Countries - April-June 2022. *N. Engl. J. Med.* **387**, 679–691 (2022). 35866746 10.1056/NEJMoa2207323

[7] Masirika, L. M. et al. Ongoing mpox outbreak in Kamituga, South Kivu province, associated with monkeypox virus of a novel Clade I sub-lineage, Democratic Republic of the Congo, 2024. *EuroSurveillence* **29**, 10.2807/1560-7917.ES.2024.29.11.2400106 (2024). 10.2807/1560-7917.ES.2024.29.11.2400106 PMC10941309 38487886

[8] Marty, A. M., Bey, C. K. & Koenig, K. L. 2024 Mpox outbreak: a rapidly evolving public health emergency of international concern: Introduction of an Updated Mpox Identify-Isolate-Inform (3I) Tool. *One Health* **19**, 100927 (2024). 39624159 10.1016/j.onehlt.2024.100927 PMC11609508

[9] Ndembi, N. et al. Evolving epidemiology of Mpox in Africa in 2024. *N. Engl. J. Med.* **392**, 666–676 (2025). 39887004 10.1056/NEJMoa2411368

[10] Nakazawa, Y. et al. A phylogeographic investigation of African monkeypox. *Viruses* **7**, 2168–2184 (2015). 25912718 10.3390/v7042168 PMC4411695

[11] Djuicy, D. D. et al. Concurrent Clade I and Clade II monkeypox virus circulation, cameroon, 1979-2022. *Emerg. Infect. Dis.* **30**, 432–443 (2024). 38325363 10.3201/eid3003.230861 PMC10902553

[12] Beiras, C. G. et al. Concurrent outbreaks of mpox in Africa-an update. *Lancet* **405**, 86–96 (2025). 39674184 10.1016/S0140-6736(24)02353-5

[13] Baliere, C. et al. Complete genome sequences of monkeypox virus from a french clinical sample and the corresponding isolated strain, obtained using nanopore sequencing. *Microbiol. Resour. Announc.* **12**, e0000923 (2023). 36971577 10.1128/mra.00009-23 PMC10112124

[14] Claro, I. M. et al. Shotgun metagenomic sequencing of the first case of monkeypox virus in Brazil, 2022. *Rev. Inst. Med. Trop. Sao Paulo* **64**, e48 (2022). 35749419 10.1590/S1678-9946202264048 PMC9217064

[15] Vakaniaki, E. H. et al. Sustained human outbreak of a new MPXV clade I lineage in eastern Democratic Republic of the Congo. *Nat. Med.* **30**, 2791–2795 (2024). 38871006 10.1038/s41591-024-03130-3 PMC11485229

[16] Chen, N. F. G. et al. Development of an amplicon-based sequencing approach in response to the global emergence of mpox. *PLoS Biol.* **21**, e3002151 (2023). 37310918 10.1371/journal.pbio.3002151 PMC10263305

[17] Licheri, M. et al. A novel isothermal whole genome sequencing approach for Monkeypox Virus. *Sci. Rep.* **14**, 22333 (2024). 39333274 10.1038/s41598-024-73613-3 PMC11437064

[18] Aja-Macaya, P. et al. A new and efficient enrichment method for metagenomic sequencing of Monkeypox virus. *BMC Genom.* **24**, 29 (2023). 10.1186/s12864-023-09114-w PMC9847149 36650445

[19] Picher, A. J. et al. TruePrime is a novel method for whole-genome amplification from single cells based on TthPrimPol. *Nat. Commun.* **7**, 13296 (2016). 27897270 10.1038/ncomms13296 PMC5141293

[20] Blagodatskikh, K. A. et al. Improved DOP-PCR (iDOP-PCR): a robust and simple WGA method for efficient amplification of low copy number genomic DNA. *PLoS ONE* **12**, e0184507 (2017). 28892497 10.1371/journal.pone.0184507 PMC5593185

[21] Mukinda, V. B. et al. Re-emergence of human monkeypox in Zaire in 1996. monkeypox epidemiologic working group. *Lancet* **349**, 1449–1450 (1997). 9164323 10.1016/S0140-6736(05)63725-7 PMC9533927

[22] Peters, J. C. An epizootic of monkey pox At Rotterdam Zoo. *Int. Zoo. Yearb.* **6**, 274–275 (1966).

[23] Hutin, Y. J. et al. Outbreak of human monkeypox, Democratic Republic of Congo, 1996 to 1997. *Emerg. Infect. Dis.* **7**, 434–438 (2001). 11384521 10.3201/eid0703.010311 PMC2631782

[24] Misu, M. et al. Nuclease-MDA for Enriching Monkeypox Virus Genomes for NGS, protocols.io, **1**, 10.17504/protocols.io.4r3l22524l1y/v1 (2026).

[25] Misu, M. et al. Terminal PCR for Amplifying the 5' and 3' Termini of the Monkeypox Virus Genome, protocols.io, **1**, 10.17504/protocols.io.36wgqdqmxvk5/v1 (2026).

[26] Shchelkunov, S. N. et al. Human monkeypox and smallpox viruses: genomic comparison. *FEBS Lett.* **509**, 66–70 (2001). 11734207 10.1016/S0014-5793(01)03144-1 PMC9533818

[27] Misu, M. et al. Rapid whole genome sequencing methods for RNA viruses. *Front. Microbiol.* **14**, 1137086 (2023). 36910229 10.3389/fmicb.2023.1137086 PMC9995502

[28] Li, H. Minimap2: pairwise alignment for nucleotide sequences. *Bioinformatics* **34**, 3094–3100 (2018). 29750242 10.1093/bioinformatics/bty191 PMC6137996

[29] Maksyutov, R. A., Gavrilova, E. V. & Shchelkunov, S. N. Species-specific differentiation of variola, monkeypox, and varicella-zoster viruses by multiplex real-time PCR assay. *J. Virol. Methods* **236**, 215–220 (2016). 27477914 10.1016/j.jviromet.2016.07.024 PMC9629046

[30] Landsberger, M., Quick, J. & Mercer, J. Coding-complete genome sequences of copenhagen and copenhagen-derived vP811 strains of vaccinia virus isolated from cell culture. *Microbiol. Resour. Announc.* **12**, e0009023 (2023). 36946721 10.1128/mra.00090-23 PMC10112197

[31] Price, M. N., Dehal, P. S. & Arkin, A. P. FastTree: computing large minimum evolution trees with profiles instead of a distance matrix. *Mol. Biol. Evol.* **26**, 1641–1650 (2009). 19377059 10.1093/molbev/msp077 PMC2693737

[32] Price, M. N., Dehal, P. S. & Arkin, A. P. FastTree 2–approximately maximum-likelihood trees for large alignments. *PLoS ONE* **5**, e9490 (2010). 10.1371/journal.pone.0009490 PMC2835736 20224823

