# Supplementary material for: Efficient whole-genome sequencing of Monkeypox virus using a novel nuclease-multiple displacement amplification enrichment method
Source: Npj Viruses. 2026 Jan 21;4:5. doi: 10.1038/s44298-026-00170-z (PMC12824382; doi:10.1038/s44298-026-00170-z)
Supplement: Supplementary file 1 — Supplementary Information [file 44298_2026_170_MOESM1_ESM.pdf]

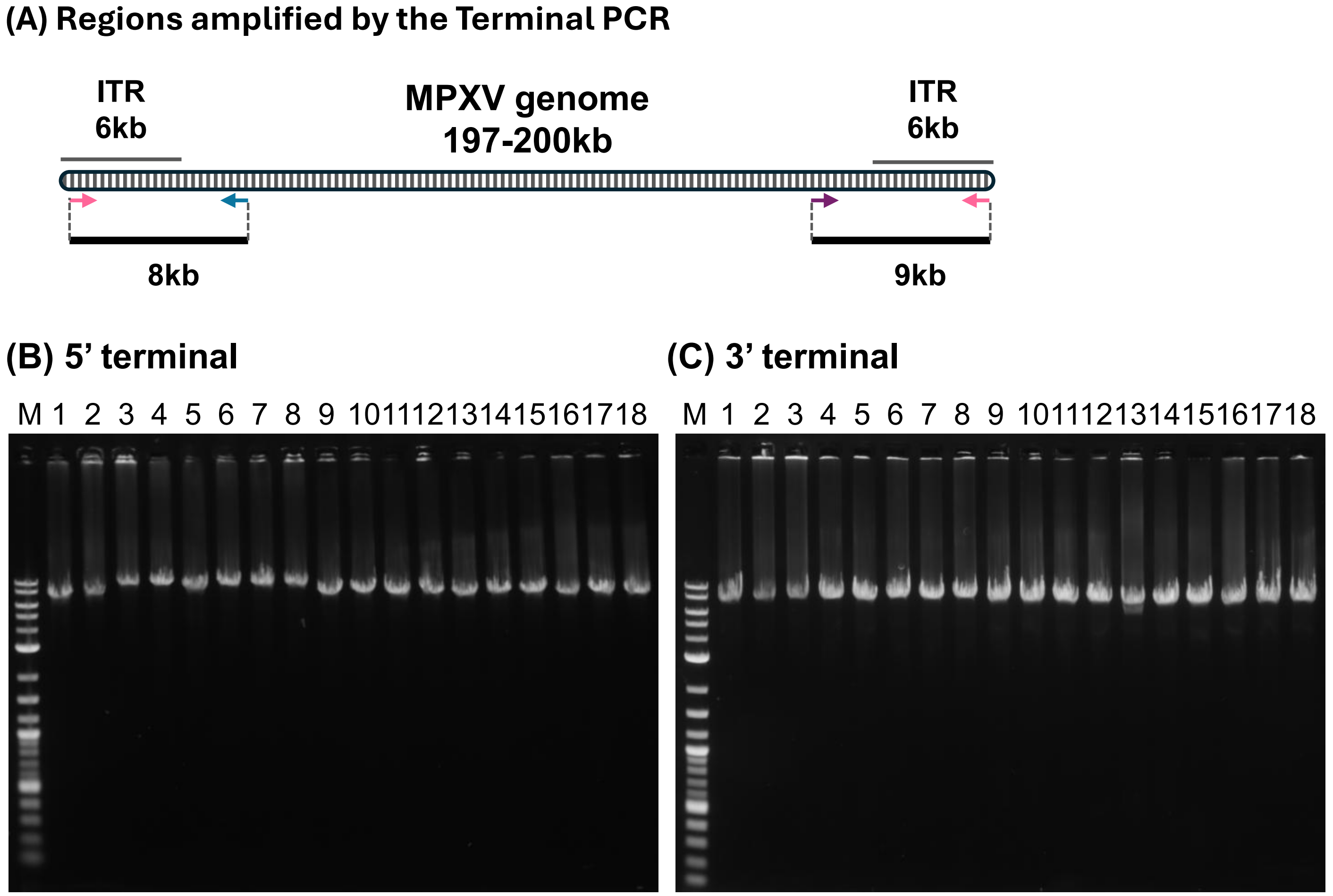

Supplementary Figure 1. Terminal PCR for the amplification of MPXV genome termini. (A) Schematic representation of the terminal regions targeted by terminal PCR. To recover terminal regions with reduced read depth, PCR is performed using primers targeting highly conserved non-repetitive regions, resulting in ~8-kb and ~9 kb-amplicons at the 5′ and 3′ ends, respectively. (B, C) Electrophoretic analysis of PCR products of the 5′ (B) and 3′ (C) terminal regions. MPXV working stocks were used as templates, and 5′ and 3′ termini were amplified separately. PCR products were analyzed on a 1% agarose gel. Lane assignments: M, 1-kb Plus DNA Ladder (NEB, N3200); 1, Zr-599; 2, Congo-8; 3, Libera; 4, Copenhagen; 5, Sierra Leone; 6, Anteater; 7, SEN-70; 8, Orangutan; 9, V96-I-071; 10, V96-I-008; 11, V96-I-003; 12, V96-I-062B; 13, V96-I-005; 14, V96-I-079A; 15, V96-I-063B; 16, V96-I-004; 17, V96-I-016; 18, V96-I-017.

Supplementary Table 1. Primer sequences used for terminal PCR

**Target**	**Forward**		**Reverse**		**Approximate product size (kb)**
	**Name**	**Sequence**	**Name**	**Sequence**	
5′ terminus	MPXV term	GTGTGACCCACGACCGTAG	MPXV_5-inner	TCCATCTCCCTCTGGACCAC	8
3′ terminus	MPXV_3-inner	AATCGTTCTCCTCGGTGTCA	MPXV term	GTGTGACCCACGACCGTAG	9

Supplementary Table 2. MPXV strains used in this study

Strain	Genotype	Source	Accession number
Congo-8	Clade Ia	Gifted by Dr. Dumbell on June 30, 1971, after four CAM passages, and further passaged one time. The July 5, 1971 stock was used.	PX119012
V96-I-003	Clade Ia	Isolated during the 1996–1997 outbreak [1]; gifted by Drs. Kenzo Kato and Tatsuo Miyamura (NIID). After three passages in LLC-MK2, stock dated April 4, 1997, was used.	PX119018
V96-I-004	Clade Ia	Same as above; after three passages in LLC-MK2, stock dated April 4, 1997, was used.	PX119019
V96-I-005	Clade Ia	Same as above; after three passages in LLC-MK2, stock dated April 4, 1997, was used.	PX119020
V96-I-008	Clade Ia	Same as above; after three passages in LLC-MK2, stock dated April 4, 1997, was used.	PX119021
V96-I-016	Clade Ia	Same as above; after two passages in LLC-MK2, stock dated October 10, 1996, was used.	PX119022
V96-I-017	Clade Ia	Same as above; after two passages in LLC-MK2, stock dated October 4, 1996, was used.	PX119023
V96-I-062B	Clade Ia	Same as above; after three passages in VeroE6, stock dated November 17, 1997, was used.	PX119024
V96-I-063B	Clade Ia	Same as above; after three passages in VeroE6, stock dated November 17, 1997, was used.	PX119025
V96-I-071	Clade Ia	Same as above; after three passages in VeroE6, stock dated November 17, 1997, was used.	PX119026
V96-I-079A	Clade Ia	Same as above; after three passages in VeroE6, stock dated November 18, 1997, was used.	PX119027
Zr-599	Clade Ia	Gifted by Dr. Nakano (CDC) on April 14, 1975; passaged twice in CAM. The November 26, 1980 stock was used.	PX119028
Anteater	Clade IIa	Gifted by Dr. Yoshiaki Ueda (NIID); passaged twice in CAM. The January 11, 1973 stock was used.	PX122227
Copenhagen	Clade IIa	Gifted by Dr. R. Gispen on April 14, 1969, after three CAM passages and further passaged four times. The June 20, 1975 stock was used.	PX119013

Liberia	Clade IIa	Gifted by Dr. Dumbell on June 30, 1971, after four CAM passages and further passaged one time. The July 5, 1971 stock was used.	PX119014
Orangutan	Clade IIa	Gifted by Dr. Yoshiaki Ueda (NIID); passaged twice in CAM. The January 11, 1973 stock was used.	PX119015
SEN-70	Clade IIa	Gifted by Dr. S.S. Kalter on March 30, 1970; passaged once in CAM. The April 3, 1970 stock was used.	PX119016
Sierra Leone	Clade IIa	Gifted by Dr. Dumbell on June 30, 1971, after three CAM passages and further passaged one time. The July 5, 1971 stock was used.	PX119017

Supplementary Table 3. Primer sequences used for qPCR.

**Target**	**Forward primer**		**Reverse primer**		**Product size (bases)**
	**Name**	**Sequence**	**Name**	**Sequence**	
LC16m8 *G9R* gene	E9L-F	TGCAACTGAACGCGTTCTTG	E9L -R	GTCATAAGGGAGTAGGCGGC	153
Rabbit *GAPDH* gene	GAPDH -F	ACTACATGGTGAGTGCTGCC	GAPDH-R	TTCCCGTTCTCAGCCTTGAC	158

Supplementary Table 4. Strains from public databases used for phylogenetic analysis

**Strain**	**Reference(s)**	**Accession #**
24MPX0198V	[2]	PP601207
24MPX0201V	[2]	PP601208
Boende_DRC_2008	[3]	KP849469
Cameroon-1990	[3,4]	KJ642618
Congo_2003_358	[5,6]	DQ011154
Congo-8	[3,7]	KJ642613
COP-58	[8-10]	AY753185
Cote d’Ivoire_1971	[3,11]	KP849470
DRC_Yandongi_1985	[12,13]	KC257460
Gabon-1988	[3,14]	KJ642619
Ikubi	[3,12]	KJ642612
Liberia_1970_184	[5,15,16]	DQ011156
MPXV-Singapore	[17]	MT903342
MPXV-WRAIR7-61	[9,18]	AY603973
Nigeria-SE-1971	[5,15,16]	KJ642617
PCH	[3,19]	KJ642616
SL-V70	[9,15,16]	AY741551
Sudan_2005_01	[13]	KC257459
USA_2003_039	[5,20]	DQ011157
USA_2003_044	[5,20]	DQ011153
UTC	[3,21]	KJ642614
V79-I-005	[22]	HQ857562
W-Nigeria	[3,23]	KJ642615
Yambuku_DRC_1985	[3,24]	KP849471
Zaire-96-I-16	[1,25]	AF380138

Supplementary Table 5. CPXV and ECTV strains used in this study

Virus	Strain	**Source**	**Accession number**
CPXV	58	Gifted by Dr. R. Gispen on September 12, 1973. The May 26, 1984 stock was used.	PX585980
CPXV	Amsterdam	Gifted by Dr. R. Gispen on September 12, 1973. The October 11, 1979 stock was used.	PX585981
CPXV	Brighton Red	Passage history unknown, before four CAM passages. The February 27, 1956 stock was used.	PX585982
ECTV	Hampstead	Passage history unknown, before four CAM passages. The July 11, 1975 stock was used.	PX585983

References of Supplementary Table 4

1 Hutin, Y. J. *et al.* Outbreak of human monkeypox, Democratic Republic of Congo, 1996 to 1997. *Emerg Infect Dis* **7**, 434-438 (2001).

2 Vakaniaki, E. H. *et al.* Sustained human outbreak of a new MPXV clade I lineage in eastern Democratic Republic of the Congo. *Nat Med* **30**, 2791-2795 (2024). https://doi.org/10.1038/s41591-024-03130-3

3 Nakazawa, Y. *et al.* A phylogeographic investigation of African monkeypox. *Viruses* **7**, 2168-2184 (2015). https://doi.org/10.3390/v7042168

4 Tchokoteu, P. F. *et al.* [Variola or a severe case of varicella? A case of human variola due to monkeypox virus in a child from the Cameroon]. *Ann Soc Belg Med Trop* **71**, 123-128 (1991).

5 Likos, A. M. *et al.* A tale of two clades: monkeypox viruses. *J Gen Virol* **86**, 2661-2672 (2005). https://doi.org/86/10/2661 [pii]

10.1099/vir.0.81215-0

6 Learned, L. A. *et al.* Extended interhuman transmission of monkeypox in a hospital community in the Republic of the Congo, 2003. *Am J Trop Med Hyg* **73**, 428-434 (2005).

7 Ladnyj, I. D., Ziegler, P. & Kima, E. A human infection caused by monkeypox virus in Basankusu Territory, Democratic Republic of the Congo. *Bull World Health Organ* **46**, 593-597 (1972).

8 von Magnus, P., Andersen, E. K., Petersen, K. B. & Andersen, A. B. A pox-like disease in cynomolgus monkeys. *Acta Pathologica Microbiologica Scandinavica* **46**, 156-176 (1959).

9 Chen, N. *et al.* Virulence differences between monkeypox virus isolates from West Africa and the Congo basin. *Virology* **340**, 46-63 (2005). https://doi.org/S0042-6822(05)00330-2 [pii]

10.1016/j.virol.2005.05.030

10 Arita, I. & Henderson, D. A. Smallpox and monkeypox in non-human primates. *Bull World Health Organ* **39**, 277-283 (1968).

11 Arita, I. & Henderson, D. A. Monkeypox and whitepox viruses in West and Central Africa. *Bull World Health Organ* **53**, 347-353 (1976).

12 Jezek, Z., Khodakevich, L. N. & Wickett, J. F. Smallpox and its post-eradication surveillance. *Bull World Health Organ* **65**, 425-434 (1987).

13 Nakazawa, Y. *et al.* Phylogenetic and ecologic perspectives of a monkeypox outbreak, southern Sudan, 2005. *Emerg Infect Dis* **19**, 237-245 (2013). https://doi.org/10.3201/eid1902.121220

14 Muller, G. *et al.* Monkeypox virus in liver and spleen of child in Gabon. *Lancet* **1**, 769-770 (1988). https://doi.org/10.1016/s0140-6736(88)91580-2

15 Foster, S. O. *et al.* Human monkeypox. *Bull World Health Organ* **46**, 569-576 (1972).

16 Lourie, B. *et al.* Human infection with monkeypox virus: laboratory investigation of six cases in West Africa. *Bull World Health Organ* **46**, 633-639 (1972).

17 Mauldin, M. R. *et al.* Exportation of Monkeypox Virus From the African Continent. *J Infect Dis* **225**, 1367-1376 (2022). https://doi.org/10.1093/infdis/jiaa559

18 McConnell, S. J., Herman, Y. F., Mattson, D. E. & Erickson, L. Monkey Pox Disease in Irradiated Cynomologous Monkeys. *Nature* **195**, 1128-1129 (1962). https://doi.org/10.1038/1951128a0

19 Milhaud, C., Klein, M. & Virat, J. Analyse d'un cas de variole du singe (monkeypox) chez le chimpanzé (Pan troglodytes). *Expériences Animales* **2**, 121-135 (1969).

20 Reed, K. D. *et al.* The detection of monkeypox in humans in the Western Hemisphere. *N Engl J Med* **350**, 342-350 (2004). https://doi.org/10.1056/NEJMoa032299

350/4/342 [pii]

21 Peters, J. C. AN EPIZOOTIC OF MONKEY POX AT ROTTERDAM ZOO. *International Zoo Yearbook* **6**, 274-275 (1966). https://doi.org/https://doi.org/10.1111/j.1748-1090.1966.tb01794.x

22 Estep, R. D. *et al.* Deletion of the monkeypox virus inhibitor of complement enzymes locus impacts the adaptive immune response to monkeypox virus in a nonhuman primate model of infection. *J Virol* **85**, 9527-9542 (2011). https://doi.org/10.1128/JVI.00199-11

23 Breman, J. G. *et al.* Human monkeypox, 1970-79. *Bull World Health Organ* **58**, 165-182 (1980).

24 Khodakevich, L., Jezek, Z. & Kinzanzka, K. Isolation of monkeypox virus from wild squirrel infected in nature. *Lancet* **1**, 98-99 (1986). https://doi.org/S0140-6736(86)90748-8 [pii]

25 Shchelkunov, S. N. *et al.* Human monkeypox and smallpox viruses: genomic comparison. *FEBS Lett* **509**, 66-70 (2001). https://doi.org/10.1016/s0014-5793(01)03144-1
